# Child and Family Outcomes After PICU Admission: Creation of an Open Access Literature Database Using a Global Team of PICU Specialists Integrated With Machine Learning

**DOI:** 10.1097/PCC.0000000000003780

**Published:** 2025-06-25

**Authors:** Rebecca E. Hay, David J. Zorko, Katie O’Hearn, Cara McQuaid, Geneviève Du Pont Thibodeau, Gonzalo Garcia Guerra, Jeremy Olivier, Laurence Ducharme-Crevier, Laurie Lee, Michael J. Del Bel, Karen Choong, James Dayre McNally

**Affiliations:** 1 Division of Pediatric Critical Care, Department of Pediatrics, University of Ottawa, Ottawa, ON, Canada.; 2 Nuffield Department of Primary Care Health Sciences, University of Oxford, Oxford, United Kingdom.; 3 Division of Pediatric Critical Care Medicine, Department of Pediatrics, McMaster University, Hamilton, ON, Canada.; 4 Children’s Hospital of Eastern Ontario Research Institute, Ottawa, ON, Canada.; 5 Division of Pediatric Critical Care Medicine, Department of Pediatrics, CHU Sainte-Justine, Université de Montréal, Montreal, QC, Canada.; 6 Division of Critical Care, Department of Pediatrics, University of Calgary, Calgary, AB, Canada.; 7 Department of Chemistry, McGill University, Montreal, QC, Canada.; 8 Department of Pediatrics, Cumming School of Medicine and Faculty of Nursing, University of Calgary, Calgary, AB, Canada.

**Keywords:** Hackathon, pediatric critical care, pediatric post-intensive care syndrome, pediatric intensive care unit outcomes, systemic and scoping review methods

## Abstract

**OBJECTIVES:**

As research examining child health outcomes after PICU admission grows, so does the need for the identification and synthesis of a large body of literature. We aimed to create an open-access scoping repository of literature describing longer-term health outcomes after PICU admission, using a large multinational team (crowdsourcing) and a machine learning (ML) algorithm.

**DATA SOURCES:**

We performed a registered scoping review (OSF DOI10.17605/OSF.IO/HE5VB; Registered November 21, 2022) using MEDLINE, Embase, CINAHL, and CENTRAL databases, 2000–2022, with no language restrictions.

**STUDY SELECTION:**

Observational or interventional studies describing outcomes of children (0–17 yr old) and their families or caregivers measured greater than 2 weeks post-PICU discharge. Titles and abstracts and full texts were initially screened by a large team of PICU healthcare workers and researchers who were recruited as part of an Evidence Hackathon event at the 2022 World Federation of Pediatric Intensive and Critical Care Societies conference. Initial screening results from 5000 citations were used to develop and validate an ML algorithm, after which a hybrid human crowdsourcing and ML approach was used to screen the remaining 11,055 studies.

**DATA EXTRACTION:**

Not applicable.

**DATA SYNTHESIS:**

Of 16,055 eligible citations, 1,301 met the criteria at full text for inclusion in the database. The screening was completed in just under 2 months while adhering to the gold standard systematic review methodology. Sensitivity for the hybrid human crowdsourcing and ML was 98%.

**CONCLUSIONS:**

A collaborative, global PICU team integrated with ML was successful in efficient and accurate large data synthesis, producing a scoping open-access database of studies reporting on post-PICU outcomes. The development of this repository has implications for future reviews, providing opportunities for networking and collaborative engagement in research. The next steps should examine database maintenance, utilization, and dissemination of research findings.

**RESEARCH IN CONTEXT**

- Research on post-PICU outcomes is a rapidly growing field, with an increasing need for synthesis of a large volume of research and clinical care data.
- At present, our field is duplicating research efforts, and there are barriers to efficient synthesis of post-PICU outcomes research.
- We used the 2022 World Federation of Pediatric Intensive and Critical Care Societies (WFPICCS) conference as an opportunity to recruit and engage members of the global PICU community to participate in synthesizing the post-PICU outcomes data, in combination with a machine learning (ML) research approach.

**WHAT THIS STUDY MEANS**

- A hybrid approach of crowdsourcing, recruiting a team of 36 qualified individuals, at the 2022 WFPICCS Evidence Hackathon and ML successfully screened 16,055 citations describing post-PICU outcomes in under 2 months with a sensitivity of 98%.
- The creation of a literature database describing post-PICU outcomes provides an opportunity to continue to streamline post-PICU outcomes research.
- Future challenges include ensuring database updates as a “living” database, and dissemination for further research output.

As PICU survivorship grows so does the demand for high-quality systematic and scoping reviews to synthesize and disseminate pediatric post-intensive care syndrome (PICS-p) knowledge (1, 2). Challenges to synthesizing what we know about PICS-p in a timely manner—particularly as the number of published studies is increasing rapidly—include the workload and resources required for, potentially, 1752 person-hours depending on the scope of a review (3). Invariably, multiple investigative teams within the pediatric critical care research community may work independently to screen large sets of citations with similar eligibility criteria for their unique research questions. A shared PICS-p data repository may expedite reviews and avoid duplicating efforts. However, the creation of such a repository would need to initially screen 10s of 1000s of citations.

Recognizing this gap and opportunity, pediatric researchers within the Canadian Critical Care Trials Group (CCCTG) therefore sought to create an open-access scoping database of child and family outcomes after PICU discharge. Our aim was, first, to engage the global pediatric critical care community, as represented by attendees at the 2022 World Federation of Pediatric Intensive and Critical Care Societies (WFPICCS) 11th Congress. Second, we aimed to use ML to assist the human team with the identification and synthesis of relevant literature. This report describes this process and the feasibility of using a combination of human crowdsourcing and machine learning (ML) as part of an “Evidence Hackathon.”

## METHODS

**Figure 1.**
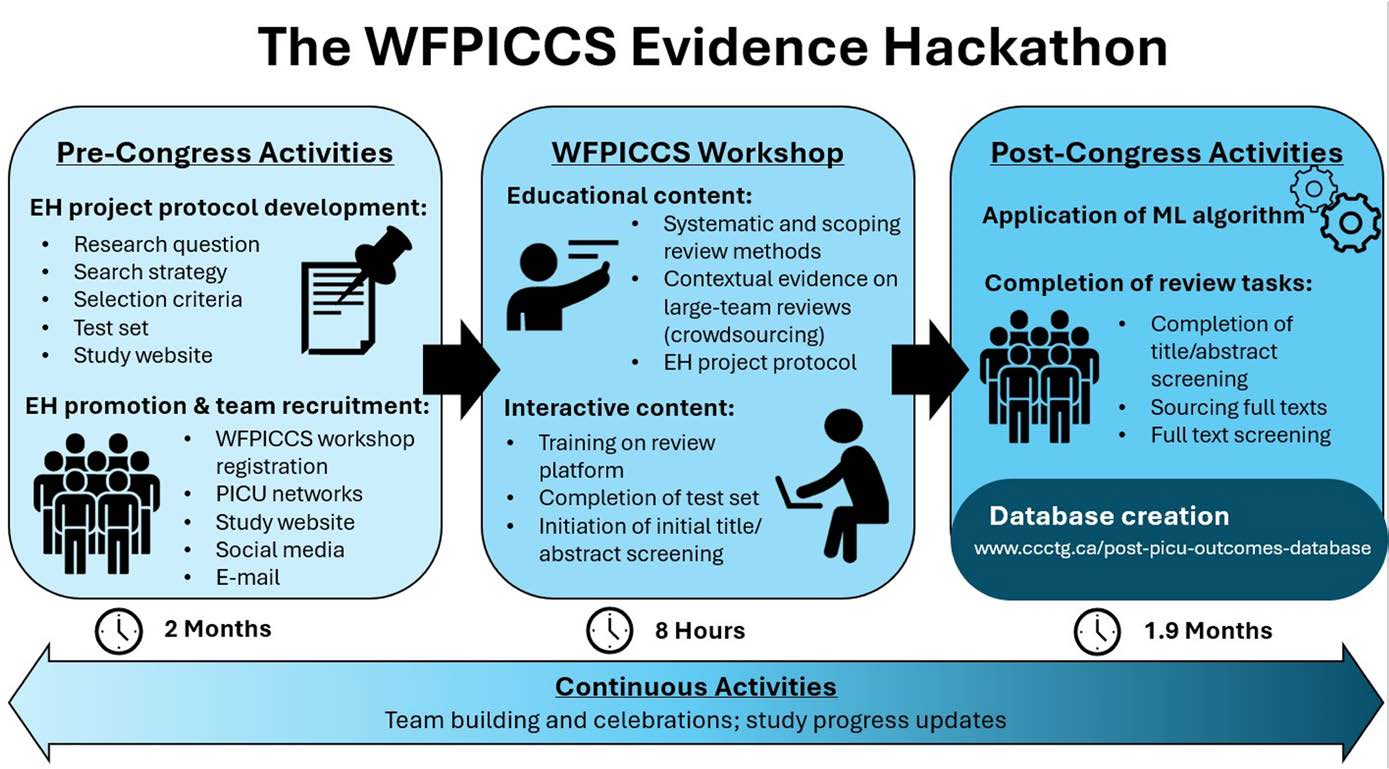
The process for the creation of a post-PICU outcomes database using crowdsourcing and machine learning (ML). Activities involved in each stage are outlined, with the time required below. The link for the open-access database is provided in “Database Creation.” EH = Evidence Hackathon, WFPICCS = World Federation of Pediatric Intensive and Critical Care Societies.

The Evidence Hackathon was designed to occur in three stages (**Fig. 1**) integrated into the 2022 WFPICCS congress held “virtually” in Cape Town, South Africa. First, before the congress, a protocol was developed for both the scoping review/database creation and a post-PICU clinic and posttraumatic stress disorder (PTSD) review from the resultant database and uploaded to Open Science Framework on November 21, 2022 (4). Multiple databases (i.e., MEDLINE, Embase, CINAHL, and CENTRAL Database) were searched from January 2000 to June 2022 using MeSH terms and keywords related to the population (e.g., pediatrics), setting (e.g., ICU), relevant admission indications or interventions, and outcomes (e.g., post-intensive care syndrome).

**Figure 2.**
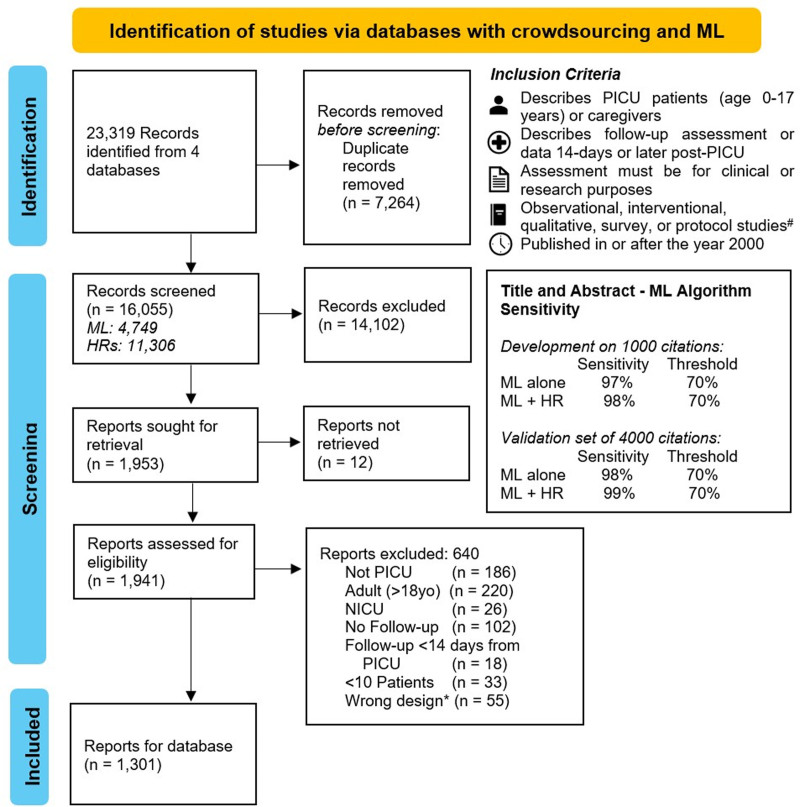
Preferred Reporting Items for Systematic Reviews and Meta-Analyses flowchart of study screening, and machine learning (ML) algorithm validation. Screened in duplicate using a hybrid crowdsourcing and ML approach validated using a set of 5000 citations, shown in the figure. Inclusion criteria for screening were: 1) PICU patients aged 0–17 yr old or caregivers, 2) citations describing follow-up assessments or data that occurred 14 days post-PICU discharge, 3) the assessment had to be for clinical or research purposes, 4) observational, interventional, qualitative, survey, or protocol study design, 5) citations published in or after the year 2000. *Study designs excluded were case reports, narrative reviews, editorials, commentaries, opinion pieces, books, news bulletins, pre-print, or conference abstracts. HR = human reviewer, ML = machine learning, NICU = neonatal ICU.

Second, at the 2022 WFPICCS congress, we planned an “Evidence Hackathon” event. Here, given the anticipated large citation set generated by the pre-congress literature searching, we planned a hybrid approach with crowdsourcing and ML (5). Crowdsourcing has been validated for reviews, whereby a large collaborative team contributes to the accelerated conduct of review tasks (6–8). WFPICCS promoted the Evidence Hackathon event before the conference, and the organizers presented it to international PICU networks. An 8-hour virtual workshop provided education and training on six steps in data gathering, including: 1) the methodology of basic scoping and systematic reviews, 2) approaches to crowdsourcing and ML, 3) using insightScope, a platform for conducting large systematic reviews, 4) the project protocol, including eligibility criteria, 5) team communication, and 6) post-PICU outcomes (Fig. 1). Individual participants recruited to the Evidence Hackathon were required to complete a test set during or shortly after the workshop. The test set contained 100 title or abstract citations with 15 true positives, requiring the correct identification of 11 of the 15 true positives (80%) to demonstrate competence. Of the 37 that attempted the test set, only 1 individual did not pass. Those who passed the test set were encouraged to start title or abstract screening. Citation inclusion criteria and reasons for exclusion are shown in **Figure 2**.

The third stage occurred after the 2022 WFPICCS congress and it involved two components. One component was focused on team collaboration and ongoing training, which happened throughout data screening with study leads (R.H., C.M.) sending regular updates and communications to the team. This follow-up included direct involvement with team members, team-building, presentation of progress metrics, and celebrating individual and project successes. Several further virtual workshops were planned and offered to address common questions or issues related to the protocol and/or software. Halfway through the screening stage, the metrics about the individual process were analyzed, and one-on-one coaching was offered as needed. At the end of database creation, all group members were invited to use the database and/or participate in planned reviews.

The other component focused on the ML algorithm, which was used to exclude low-probability citations during title/abstract screening. The ML algorithm used a numerical statistic called Term Frequency-Inverse Document Frequency (TF-IDF); this statistic reflects how important a word is to a document (or documents) by considering both its frequency and its rarity within a dataset (5, 9). We developed the TF-IDF algorithm using the text prepared related to the goals of the project, eligibility criteria, and true positive papers. Ultimately, the ML algorithm was developed and validated on a total subset of 5000 citations that had been fully screened independently and in duplicate by human review, of both title or abstract and the full text. For further details on the machine-learning development and validation, see Figure 2. The ML is publicly available (see https://github.com/Insight-Scope/wfpiccs-public-ml).

## RESULTS

After citation deduplication, we identified 16,055 unique citations that were used for screening (Fig. 2). The Evidence Hackathon team had 36 members, originating from 10 countries and 5 continents. Within the team, 7 of 36 were medical trainees (i.e., medical students, residents, or fellows), 17 of 36 were PICU attending physicians, 6 of 36 were researchers, and 2 of 36 were PICU nurses and researchers. Also, of these 36 members, there was at least 1 PICU individual from each of the following backgrounds: 1) nurse practitioner and researcher, 2) physiotherapist, 3) pharmacist, and 4) ICU-patient partner.

Screening began during the Evidence Hackathon (July 12, 2022), and was completed on September seventh, 2022, that is, a total of 56 days. After completing the screening of titles or abstracts, with any conflict resolution, there were 1941 citations that required full-text review, and of these were 1301 items that met eligibility for the scoping database (Fig. 2). The resulting database of post-PICU outcome studies is open access and can be accessed publicly, free of charge, on the CCCTG website (see https://www.ccctg.ca/post-picu-outcomes-database).

Overall, the hybrid crowdsourced and ML algorithm achieved a sensitivity of 98%. Considering an average time of 30–60 seconds to assess a citation’s title or abstract (10), the ML algorithm’s completion of 4749 records (9498 assessments) saved the team an estimated 79–158 hours of work at the title or abstract screening stage while maintaining sensitivity. Figure 2 provides details on the application of the ML algorithm to the citation set (i.e., the number of records screened by humans and machines).

## DISCUSSION

In this report, we have described the creation of an open-access scoping database of post-PICU outcomes, which was efficiently and accurately achieved in just under 2 months using 16,055 citations while following gold standard review methodology. We demonstrated a feasible data-driven method to conduct large citation synthesis, through the integration of an ML algorithm with a global team of PICU health and research professionals. To our knowledge, this was the first PICU Evidence Hackathon event conducted through a conference to engage a global research collaborative. The database can now be used to conduct future knowledge syntheses in this area.

Broad-scoping research questions have been challenging to address in systematic reviews when using the traditional approach of processing citations with a small team. Conferences congregate an international community, with research methods and content experts in a field, providing an arena to assemble a large and diverse team. We found that the 2022 WFPICCS Evidence Hackathon provided a unique opportunity to conduct a large scoping review. The team recruited included individuals from multiple institutions and multiple areas of the world, which was an advantage since we were able to source a significant number of full texts (99.4%) without any language limitation. Furthermore, integration with ML was instrumental in helping us to reduce the human workload by 48% at the title or abstract screening stage. This integrated approach allowed an accurate and efficient review of 16,055 citations in 2 months. By comparison, typical times to complete a large scoping review may take up to 20 months (11).

The post-PICU outcomes database can serve future investigators to more efficiently conduct high-quality and timely knowledge syntheses on several outcomes while avoiding laborious duplication of efforts. Using the database, an expedited citation screening process may be used for new post-PICU outcomes research questions. For example, a focused set of search criteria may be applied related to a specific concept such as PTSD, and the search output may be screened according to title or abstract, and/or full text using the investigators’ criteria. As all the studies in the database have been screened in duplicate through two stages, future investigators may alternatively choose to integrate single-reviewer assessments for their own focused project. Single-reviewer assessments retain sensitivity, and this approach will decrease workload when applied to records that are ineligible by multiple criteria (12). To date, two literature reviews have used the database with the same team, maintaining networking and collaboration for years after the 2022 event. The meta-analysis of prevalence and population-level factors contributing to PTSD in PICU survivors was published in April 2025 (13). A second scoping review protocol about the characteristics of PICU follow-up clinics using the database is ongoing (14).

Regarding medical education, we know from a scoping review of the literature published 2008–2022 that conference evaluations show eight domains targeted by attendees, including education-networking, education-learning, impact, scholarship, value-satisfaction, logistics, equity-diversity-inclusivity, and career influences (15). The inclusion of an Evidence Hackathon in a conference may enhance attendees’ experience and fulfill some of these domains. For example, continued participation in WFPICCS (16), as we have done, will hopefully provide more opportunities for networking across the globe, connecting PICU trainees and leaders interested in a common topic. To better understand this potential benefit, future evaluation through the survey is planned to describe Evidence Hackathon participant perceptions and experience.

Future considerations include ongoing database updates, cost barriers to participation, and dissemination. Since the database creation in 2022, an update is currently underway to maintain citation recency and maintain a “living” database. Individuals from the original 2022 WFPICCS Evidence Hackathon group have stayed engaged and participated in this update, as well as contributed to two further reviews using the database (13, 14). A second Evidence Hackathon at a future conference may be an effective method to ensure ongoing recruitment and collaboration of like-minded individuals. In this event, consideration should be given to removing or reducing financial barriers to conference participation, which should improve inclusivity and accessibility for all individuals. Regarding dissemination, while the database is publicly available and Evidence Hackathon group members may have used the database for reviews, we would ideally want more members of the PICU community to use this resource. Currently, dissemination has been through word of mouth and via the CCCTG website (see https://www.ccctg.ca/post-picu-outcomes-database). How to best share global research resources may be an important next step as the international PICU community continues to grow.

## CONCLUSIONS

Efficient and accurate large data synthesis is possible through integrating crowdsourcing of a diverse and collaborative team and an ML algorithm. Conferences provide a useful venue to engage interested and skilled individuals, as demonstrated here in the 2022 WFPICCS Evidence Hackathon. The new database of post-PICU outcomes studies can now be used by investigators to conduct efficient systematic and scoping reviews in this field.

## ACKNOWLEDGMENTS

We thank Margaret Sampson, MLIS, PhD, AHIP (CHEO Research Institute) for developing the electronic search strategies. We thank the World Federation of Pediatric Intensive and Critical Care Societies for providing a platform to recruit and bring together our team for the 2022 Evidence Hackathon event.
